# So, we are now talking dentistry – And it will indeed be a mouthful

**DOI:** 10.1016/j.fhj.2026.100569

**Published:** 2026-09-30

**Authors:** Jasmine Ho

**Affiliations:** Northwick Park Hospital, London North West University Healthcare NHS Trust, London, United Kingdom

When I was invited to serve as guest editor for this special issue, I enthusiastically welcomed the opportunity. As a dual-qualified oral and maxillofacial surgeon, I have long been aware of the subtle yet consequential differences between medicine and dentistry, and of the unique position my specialty occupies in bridging these two domains. I therefore saw this special issue as an ideal opportunity to introduce aspects of dentistry to a predominantly medical readership, with the hope of demystifying dental service provision, education, and the patient experience.

Traditionally, OMFS surgeons were dentally qualified first before pursuing medical training to be eligible for OMFS training. So, rather unconventionally, I first qualified in medicine and then I decided to commit to OMFS as a career path during my surgical training. This meant returning to undergraduate dental school later in my career, entering the programme as a mature student after many years of medical postgraduate clinical practice. This experience was illuminating in ways I had not anticipated. It challenged assumptions formed during my medical training and revealed gaps that only became apparent once I stepped into the dental curriculum. From this journey, four observations emerged, and they guided the selection of topics for this special issue.

1. **The medical-dentistry divide**: My first realisation was how little most medical practitioners, including myself, know about the mouth, teeth, and oral health. Even with the informal exposure I gained during early OMFS training, I was struck by the breadth of knowledge I still lacked. Additionally, I had to re-learn how to speak ‘dentistry’, which had distinctly different terminologies from medicine. The impact of this can contribute to difficulties in relating to each other. Dr Victoria Sampson discusses in her article that the ‘language barrier’ between medicine and dentistry can contribute to fragmented care and a persistent tendency to overlook the mouth as a vital organ in maintaining systemic health. Her article also highlights the need for medical professionals to understand the importance of the oral microbiome for systemic health, which is often not covered in medical school.^1^
2. **Curriculum differences**: Dental education places notable emphasis on business acumen, ethics, and leadership, reflecting the reality that many dentists will work in the private sector, in stark contrast to my medical training. Whether similar content should be incorporated into medical curricula is debatable. However, this difference in curriculum content prompted a broader consideration of whether aspects of dentistry should be taught in medical school, particularly given point 1 above. My colleague Clarissa Hjalmarsson and I attempted to explore this in our debate article: *Dental health is so crucial that it should be in the medical school curriculum.*^2^
3. **Innovation adoption in dentistry**: Dentistry embraces innovation with remarkable speed. Undergraduate students in dental hospitals routinely encounter new materials and technologies as they emerge. Yet this raises important questions about equitable access, particularly within the NHS. In her *Medicine 2050* article, Dr Sara Hurley argues compellingly for why technological integration is essential for the future of dentistry.^3^
4. **NHS dentistry**: The governance and funding of NHS dentistry proved far more complex than I had appreciated. Varying fee structures remain elusive to the public, and recent contract reforms have left even dental professionals uncertain about their long-term implications or sustainability. Understanding these complexities provides important context for the pressures currently facing frontline NHS dentistry.

For broader context, the UK government and WHO recognise that oral disease is the most prevalent noncommunicable disease globally.^4^, ^5^ Poor oral health affects daily functioning, and its consequences for children are particularly concerning. Tooth decay remains the leading cause of hospital admissions among children aged five to nine in the UK.^6^ This is something which my colleagues and I have personally witnessed as OMFS providers in secondary care settings. Dental infections in young children can affect their physical health and potential intellectual and developmental outcomes.^7^ It is thus difficult not to ask how such preventable conditions have been allowed to escalate to such worrying statistics. Healthwatch UK reports persistent barriers to accessing NHS dentistry in its patient experience surveys: four in five people struggle to obtain care; 73% report difficulty accessing care when needed; and nearly a quarter avoid dental visits due to the cost.^8^ Although the 2026 NHS dentistry reforms aim to address these issues, the impact of the new dentistry budget on patients and providers remains uncertain.^4^

Against this backdrop, I approached colleagues to contribute perspectives on the future of NHS dentistry. Their response revealed unexpected hesitancy. Many feared professional repercussions for speaking about systemic issues. Those working exclusively in private practice felt too removed from current NHS realities to comment meaningfully. I am therefore particularly grateful to **Dr Sara Hurley** for her insightful *Medicine 2050* article discussing her vision of dentistry in the future.^3^ As former Chief Dental Officer for England, her description of the dichotomous futures for dentistry in England provides a stark reality check, signalling the need for significant investment in finance and workforce, a shift in political agenda and policy changes, and a desperate need for effective leadership to drive this process towards a meaningful future. Her five commissioning imperatives provide a constructive framework for positive stimuli necessary to drive change.

The gut microbiome’s role in systemic health is well established^9^; the significance of the oral microbiome has only recently emerged as an equally important area of research. Evidence increasingly demonstrates that oral health influences far more than its local structures. Microbiome changes in periodontal disease have been linked to cardiovascular and metabolic conditions, and research into the oral microbiome–brain axis is gaining momentum, revealing associations with neurological disease and cognitive decline.^10^, ^11^, ^12^ The importance of oral health and its broader impact on overall health is highlighted in **Dr Victoria Sampson’s** article on oral microbiome.^1^ Emerging research also implicates the oral microbiome in oral carcinogenesis.^13^ With the increasing incidence of colon cancer in the younger population, similar trends are beginning to surface in oral cancer, with an unexpected increase in incidence in a subgroup of perceived low-risk patients – the young, non-smoking and non-drinking (NSND) individuals.^14^, ^15^ Researchers are only beginning to unravel this phenomenon, and more evidence points to significant changes in their oral microbiome. Her article challenges clinicians to adopt a more holistic approach and understand the crucial role of oral hygiene in relation to systemic health, making it a worthwhile read. Perhaps knowing this can better prepare medical professionals to promote oral health during their consultations.

This issue also highlights Special Care Dentistry (SCD), a discipline that quietly supports some of the most vulnerable NHS patients. From my own experience of the SCD service as a dental student, I was surprised by the manner in which they serve the community. These are dentists who would conduct home visits with mobile dental kits, provide care for homeless populations in mobile units, and deliver school-based oral health promotion to help keep dental care accessible and available to vulnerable groups. Hence, **Dr Yvonne Rooney’s** article offers a unique and insightful overview of this field of dentistry and its current challenges in service delivery.^16^ I hope that through this introduction SCD to the FHJ’s readership, it will raise awareness of their provisions and benefit those who require access to these essential services.

An often-misunderstood specialty is oral maxillofacial surgery (OMFS). Often perceived as the hospital’s ‘out-of-hours dentists’, the scope of practice encompasses far more, including facial trauma, orthognathic surgery, and head and neck oncology and facial skin cancer. The mandated dual-qualification requirement for OMFS training in the UK, parts of Europe, and the United States has produced a uniquely skilled workforce, though it also unfortunately contributes to confusion about the specialty’s identity. In the UK, despite OMFS being part of the medical and surgical fraternity, exposure to OMFS in medical school is inconsistent, as evidenced by a survey of UK medical undergraduates.^17^ In the debate article**,** both **Ms Clarissa Hjalmarsson and I** examine whether aspects of dental education should be incorporated into the medical curricula, a timely question for current medical education.^2^

Finally, I am deeply grateful to **Mr Neil Shah**, OMFS consultant colleague, for sharing his deeply personal experience as a head and neck cancer patient. His reflection offers rare insight into what it means to inhabit and convey experiences from both sides of the consultation room. The ‘Triad of Care’ (delay, certainty, and dignity) he describes provides a powerful framework for understanding the patient journey.^18^ It is not uncommon in medicine for clinicians to become desensitised as a coping mechanism. His account serves as a gentle reminder of our fundamental privilege as healthcare providers in our patients’ journey: to care for patients at the most vulnerable moments of their lives, and to help them make the best of it.

In a healthcare landscape where the mouth is still too often regarded as an afterthought, the following articles will demonstrate why dentistry should play a more integral role in holistic patient care. By examining the emerging significance of the oral microbiome and the complex realities shaping the future of NHS dentistry and medical education, they will provide an insightful exploration of where medicine and dentistry converge and where both should go next.

## Funding

This research did not receive any specific grant from funding agencies in the public, commercial, or not-for-profit sectors.

## Declaration of Competing Interest

The authors declare that they have no known competing financial interests or personal relationships that could have appeared to influence the work reported in this paper.
